# Effects of Na^+^ Current and Mechanogated Channels in Myofibroblasts on Myocyte Excitability and Repolarization

**DOI:** 10.1155/2016/6189374

**Published:** 2016-11-17

**Authors:** Heqing Zhan, Jingtao Zhang, Jialun Lin, Guilai Han

**Affiliations:** ^1^Department of Information Technology, Hainan Medical University, Haikou 571199, China; ^2^Cardiac Arrhythmia Center, Fuwai Hospital, National Center for Cardiovascular Diseases, Beijing 100037, China

## Abstract

Fibrotic remodeling, characterized by fibroblast phenotype switching, is often associated with atrial fibrillation and heart failure. This study aimed to investigate the effects on electrotonic myofibroblast-myocyte (Mfb-M) coupling on cardiac myocytes excitability and repolarization of the voltage-gated sodium channels (VGSCs) and single mechanogated channels (MGCs) in human atrial Mfbs. Mathematical modeling was developed from a combination of (1) models of the human atrial myocyte (including the stretch activated ion channel current, *I*_SAC_) and Mfb and (2) our formulation of currents through VGSCs (*I*_Na_Mfb_) and MGCs (*I*_MGC_Mfb_) based upon experimental findings. The effects of changes in the intercellular coupling conductance, the number of coupled Mfbs, and the basic cycle length on the myocyte action potential were simulated. The results demonstrated that the integration of *I*_SAC_, *I*_Na_Mfb_, and *I*_MGC_Mfb_ reduced the amplitude of the myocyte membrane potential (*V*_max_) and the action potential duration (APD), increased the depolarization of the resting myocyte membrane potential (*V*_rest_), and made it easy to trigger spontaneous excitement in myocytes. For Mfbs, significant electrotonic depolarizations were exhibited with the addition of *I*_Na_Mfb_ and *I*_MGC_Mfb_. Our results indicated that *I*_SAC_, *I*_Na_Mfb_, and *I*_MGC_Mfb_ significantly influenced myocytes and Mfbs properties and should be considered in future cardiac pathological mathematical modeling.

## 1. Introduction

Recent studies have demonstrated a correlation between atrial fibrillation (AF) and fibrotic remodeling, specifically fibrosis [1–3]. However, their relationship has not been fully understood. Changes have taken place during the process of fibrotic remodeling in terms of gap junction remodeling [4], the deposition of excess collagen [5], and fibroblast phenotype switching [2, 6], which determines the degree of AF initiation and maintenance in atrial fibrosis in AF subjects [7].

Different currents have been identified in cardiac fibroblasts by recent electrophysiological studies, including the currents through potassium channels [8, 9], the nonselective transient receptor potential cationic channel subfamily M member 7 (TRPM7) [10], voltage-gated sodium channels (VGSCs) [11], chloride channels [12], single mechanogated channels (MGCs) [13], and voltage-dependent proton currents [14]. Therefore, fibroblasts are no longer considered as nonexcitable cells. As one of the main characteristics of fibrotic remodeling, fibroblasts proliferation and differentiation into myofibroblasts (Mfbs) at the cellular level have been shown to play an important role in cardiac pathological status [15, 16]. During fibroblasts differentiation, significantly increased potassium channels and TRPM7 have been reported [10, 17]. Specifically, with the measurement of the neoexpression of rapid Na^+^ currents through VGSCs during the process, 75% of the Mfbs derived from the culture of human atrial fibroblasts expressed Na^+^ current between 8 and 12 days. After 12 days, 100% of the Mfbs expressed Na^+^ current [11]. In addition, there is strong evidence that this Na^+^ current is generated by Na_v_ 1.5 *α*-subunit, a typical VGSC to produce Na^+^ current in cardiomyocytes [11].

Recent studies have also suggested that MGCs were modulated by mechanical deformations of fibroblasts, which may contribute to the cardiac mechanoelectrical feedback under both physiological and pathophysiological conditions [18, 19]. As myocytes start contracting, the interposed fibroblasts are mechanically compressed. The membrane potential of fibroblasts is depolarized by ionic currents through MGCs [13, 18].

Computational models of atrial fibrosis have been used to investigate the relationship between fibrotic remodeling and AF. For gap junction remodeling, simulation results showed that increased anisotropy led to sustained reentrant activity [20]. For collagen deposition, it has been demonstrated that patchy distributions of collagen were responsible for atrial conduction disturbances in failing hearts [21, 22]. For fibroblast proliferation and phenotype switching, studies indicated that coupling of fibroblasts to atrial myocytes resulted in shorter duration of the action potential (APD), slower conduction, and the development of AF [23, 24]. Taking Mfbs into account, other studies combined all the above-mentioned elements in the left atrial model to examine the underlying mechanisms of AF initiation and indicated that Mfbs exerted electrotonic influences on myocytes in the lesions [1, 25]. However, all these published simulation studies have not considered the following: first, the stretch activated ion channel current (*I*_SAC_) in myocytes, which significantly influences the electrophysiological characteristics of cardiac myocytes under stretching [26, 27]; second, the currents through VGSCs (*I*_Na_Mfb_) and MGCs (*I*_MGC_Mfb_) in Mfbs, which could influence Mfb properties and contribute to mechanoelectrical coupling in cardiac pathologies [11, 13].

The present study aimed to investigate the combinational role of *I*_SAC_, *I*_Na_Mfb_, and *I*_MGC_Mfb_ in the excitability and repolarization of cardiac myocytes by Mfb-myocyte (Mfb-M) coupling. Specifically, a new formulation of *I*_Na_Mfb_ and *I*_MGC_Mfb_ based on experimental data will be combined with models of human atrial myocyte (including *I*_SAC_) and Mfb. Simulation results of human atrial myocyte action potential (AP) dynamics from different gap-junctional conductances (*G*_gap_), number of coupled Mfbs, and basic cycle lengths (BCLs) will be compared.

## 2. Materials and Methods

**Figure 1:**
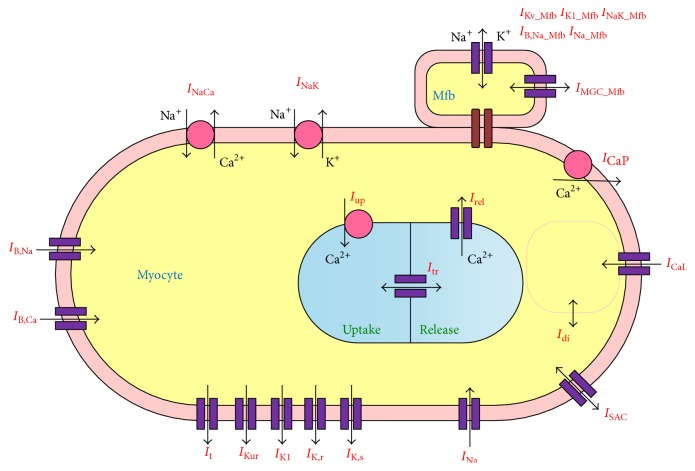
Schematic representation of the mathematical model of Mfb-M coupling.

The framework of the coupled Mfb-M model was developed from Maleckar et al.'s model of the human atrial myocyte [28] (including *I*_SAC_ equations described by Kuijpers et al. [26]), MacCannell et al.'s model of the human cardiac Mfb [24], and our new formulation of *I*_Na_Mfb_ and *I*_MGC_Mfb_ based on experimental findings from Chatelier et al. [11] and Kamkin et al. [13].  Figure 1 is a schematic of the Mfb-M coupling model. An overview of the simulations is described as follows.

### 2.1. Mfb-M Coupling

According to [24], the differential equations for the membrane potential of cardiac Mfb and myocyte are given by

(1) dVMfb,idt=−1Cm,MfbIMfb,iVMfb,i,t+Ggap⁡VMfb,i−VM,dVMdt=−1Cm,MIMVM,t+∑i=1nGgap⁡VM−VMfb,i,

where *V*_Mfb,i_ and *V*_M_ represent the transmembrane potential of the *i*th coupled Mfb and the human atrial myocyte, respectively; C_m,Mfb_ and C_m,M_ represent the membrane capacitance of the Mfb and the myocyte, respectively; *I*_Mfb,i_ and *I*_*M*_ represent the transmembrane current of the *i*th coupled Mfb and the human atrial myocyte, respectively; and *G*_gap_ represents the gap-junctional conductance, which varies between 0.5 and 8 nS in individual simulations based on previous measurements [19, 29]. A negative *I*_gap⁡_ (i.e., *G*_gap_ (*V*_Mfb,*i*_ − *V*_*M*_)) indicates that the current is flowing from the myocyte into the *i*th Mfb, and *n* is the total number of coupled Mfbs.

### 2.2. Mathematical Model of the Human Atrial Myocyte

The mathematical model from Maleckar et al. was implemented in this study [28], which was based on experimental data and has correctly replicated APD restitution of the adult human atrial myocyte. To describe the influence of stretch on myocyte AP, the original model from Maleckar et al. was modified with the total ionic current of myocyte (*I*_*M*_) given by

(2) IMVM,t=INa+ICaL+It+IKur+IK1+IK,r+IK,s+IB,Na+IB,Ca+INaK+ICaP+INaCa+ISAC−IStim,

where *I*_Na_ is fast inward Na^+^ current; *I*_CaL_ is L-type Ca^2+^ current; *I*_t_ is transient outward K^+^ current; *I*_Kur_ is sustained outward K^+^ current; *I*_K1_ is inward rectifying K^+^ current; *I*_K,r_ is rapid delayed rectifier K^+^ current; *I*_K,s_ is slow delayed rectifier K^+^ current; *I*_B,Na_ is background Na^+^ current; *I*_B,Ca_ is background Ca^2+^ current; *I*_NaK_ is Na^+^-K^+^ pump current; *I*_CaP_ is sarcolemmal Ca^2+^ pump current; *I*_NaCa_ is Na^+^-Ca^2+^ exchange current; *I*_SAC_ is stretch activated current; and *I*_Stim_ is stimulated current.

On the basis of experimental observations [30], it has been reported by Kuijpers et al. that *I*_SAC_ in atrial myocytes is permeable to Na^+^, K^+^, and Ca^2+^ [26], which is defined as

(3) $$\begin{array}{c} I_{\text{SAC}}=I_{\text{SAC,Na}}+I_{\text{SAC,K}}+I_{\text{SAC,Ca}}, \end{array}$$

where *I*_SAC,Na_, *I*_SAC,K_, and *I*_SAC,Ca_ represent the contributions of Na^+^, K^+^, and Ca^2+^ to *I*_SAC_, respectively. These currents were defined by the constant-field Goldman-Hodgkin-Katz current equation [26].

To introduce the effect of *I*_SAC_ on intracellular Na^+^, K^+^, and Ca^2+^ concentrations ([Na^+^]_*i*_, [K^+^]_*i*_, and [Ca^2+^]_*i*_), we replaced equations of [Na^+^]_*i*_, [K^+^]_*i*_, and [Ca^2+^]_*i*_ in Maleckar et al.'s model [28] by

(4) dNa+idt=−INa+IB,Na+3INaK+3INaCa+ISAC,NaVoliF,dK+idt=−It+IKur+IK1+IK,s+IK,r−2INaK+ISAC,KVoliF,dCa2+idt=−−Idi+IB,Ca+ICaP−2INaCa+Iup−Irel⁡+ISAC,Ca2.0VoliF−dOdt,

where *F* is Faraday's constant; Vol_*i*_ is cytosolic volume; *I*_di_ is Ca^2+^ diffusion current from the diffusion-restricted subsarcolemmal space to the cytosol; *I*_up_ is sarcoplasmic reticulum Ca^2+^ uptake current; *I*_rel⁡_ is sarcoplasmic reticulum Ca^2+^ release current; and* O* is buffer occupancy.

### 2.3. Electrophysiological Model of the Human Atrial Mfb

The electrophysiological model of the human atrial Mfb from MacCannell et al. [24] was used in the present study. It included time- and voltage-dependent K^+^ current (*I*_K*v*_Mfb_), inward rectifying K^+^ current (*I*_K1_Mfb_), Na^+^-K^+^ pump current (*I*_NaK_Mfb_), and Na^+^ background current (*I*_B,Na_Mfb_).

In addition, *I*_Na_Mfb_ and *I*_MGC_Mfb_ were added in the Mfb model. According to the published experimental data [9], the Mfb transmembrane potential *V*_Mfb_ and *C*_*m*,Mfb_ of −47.75 mV and 6.3 pF were used.

For *I*_Na_Mfb_, the steady-state value of activation gating variable for Na^+^ current (${\overset{-}{m}}_{\text{Mfb}}$) was obtained by fitting the experimental data from Chatelier et al. [11] with a Boltzmann function using Origin software:

(5) $$\begin{array}{c} y=\frac{A_{1}-A_{2}}{1+e^{\left(V_{\text{Mfb}}-V_{\text{0.5}}\right)/k}}+A_{2}, \end{array}$$

where *V*_Mfb_ is the Mfb potential ranging from −120 mV to 40 mV in 10 mV increments and *y* is the corresponding value of ${\overset{-}{m}}_{\text{Mfb}}$ at each *V*_Mfb_. *A*_1_ and *A*_2_ are fitting coefficients and *V*_0.5_ and *k* represent the half-maximum voltage of activation and the Boltzmann steepness coefficient, respectively.

After the data were fitted with a Boltzmann function, *A*_1_ and *A*_2_ were −0.0102 and 1.0063, respectively.* V*_0.5_ and* k* were −42.1 mV and 10.53, respectively. The equation of ${\overset{-}{m}}_{\text{Mfb}}$ was then expressed as

(6) $$\begin{array}{c} {\overset{-}{m}}_{\text{Mfb}}=\frac{-0.0102-1.0063}{1.0+e^{\left(V_{\text{Mfb}}+42.1\right)/10.53}}+1.0063. \end{array}$$

The steady-state value of inactivation gating variable for *I*_Na_Mfb_ (${\overset{-}{j}}_{\text{Mfb}}$) was also fitted with the Boltzmann function, with *A*_1_ and *A*_2_ of 1.04 and 0.004 and *V*_0.5_ and *k* of −84.82 mV and 9.4, respectively. The equation of ${\overset{-}{j}}_{\text{Mfb}}$ was given as

(7) $$\begin{array}{c} {\overset{-}{j}}_{\text{Mfb}}=\frac{1.04-0.004}{1.0+e^{\left(V_{\text{Mfb}}+84.82\right)/9.4}}+0.004. \end{array}$$

As suggested by Chatelier et al. that *I*_Na_Mfb_ was generated by the same Na_v_ 1.5 which produced Na^+^ currents in the atria and ventricle of the adult human heart [11], the equations of activation time constants in the model of Courtemanche et al. [31] were used to describe the activation time constant for *I*_Na_Mfb_ (*τ*_*m*_Mfb__). The equations are given by

(8) αmMfb=A1VMfb+Vx1−eA2VMfb+Vx,

(9) βmMfb=A3e−VMfb/A4,

(10) τmMfb=1αmMfb+βmMfb,

where *V*_Mfb_ are original values, ranging from −40 mV to 30 mV in 10 mV increments and *τ*_*m*_Mfb__ is the corresponding value at each *V*_Mfb_. *α*_*m*_Mfb__and *β*_*m*_Mfb__are extrapolated rate coefficients. After the data were fitted with these equations, *A*_1_ to *A*_4_ were 0.0077, −0.18, 0.004, and 11.98, and *V*_*x*_ was 68.19 mV.

Equation (8) requires evaluation of a limit to determine the value at membrane potential for which its denominator is zero. Equations (8) and (9) are therefore expressed as follows:

(11) αmMfb=0.0077VMfb+68.191.0−e−0.18VMfb+68.190.0433,if VMfb=−68.19βmMfb=0.004e−VMfb/11.98.

Similarly, the equations of inactivation time constant (*τ*_*j*_Mfb__) were as follows:

(12) αjMfb=−14.5e0.17VMfb+1.8e1.56VMfb·VMfb+35.731.0+e3.31VMfb+3.860,if  VMfb≥−40.0,βjMfb=5.05×10−5e−0.0995VMfb1.0+e−0.01VMfb−84.020.11e−4.13×10−4VMfb1.0+e−0.09VMfb+42.44,if  VMfb≥−40.0,τjMfb=1αjMfb+βjMfb.

The model of *I*_Na_Mfb_ was modified from the equation described by Luo and Rudy [32], as follows:

(13) INa_Mfb=g−Na,MfbmMfbjMfb0.12VMfb−ENa,Mfb,ENa,Mfb=RTFlog⁡Na+c,MfbNa+i,Mfb,

where ${\overset{-}{g}}_{\text{Na,Mfb}}$, the maximum conductance of *I*_Na_Mfb_, was 0.756 nS; *E*_Na,Mfb_ is the Nernst potential for Na^+^ ions; [Na^+^]_c,Mfb_, the Mfb extracellular Na^+^ concentration, was 130.011 mmol/L; [Na^+^]_i,Mfb_, the Mfb intracellular Na^+^ concentration, initial value was 8.5547 mmol/L; *m*_Mfb_ and *j*_Mfb_ were the activation and inactivation parameters, respectively. In order to conform to the experiment data [11], *j* was modified to be* j* ^0.12^. Equations of time dependence were given by

(14) dmMfbdt=m−Mfb−mMfbτmMfb,djMfbdt=j−Mfb−jMfbτjMfb.

For *I*_MGC_Mfb_, the equation based on experimental results from Kamkin et al. [13] was given by

(15) $$\begin{array}{c} I_{\text{MGC\_Mfb}}={\overset{-}{g}}_{\text{MGC,Mfb}}\cdot \left(\;V_{\text{Mfb}}-E_{\text{MGC,Mfb}}\;\right), \end{array}$$

where ${\overset{-}{g}}_{\text{MGC,Mfb}}$, the maximum conductance of *I*_MGC_Mfb_, was 0.043 nS and *E*_MGC,Mfb_, the reversal potential of MGCs, was close to 0 mV [13].

### 2.4. Simulation Protocol

Simulations were carried out with (1) two Mfbs coupled to a single atrial myocyte in which *G*_gap_ varied between 0.5 and 8 nS and (2) one and four identical Mfbs coupled to a single atrial myocyte at constant *G*_gap_ of 3 nS. Next, in order to investigate the role of BCL in myocyte AP, the coupled system was paced with BCLs from 100 to 2000 ms for each *G*_gap_ (0.5 or 8 nS) and for each number of coupled Mfbs (1 or 4). The resting myocyte membrane potential (*V*_rest_), the amplitude of the myocyte membrane potential (*V*_max_), and APD at 90% repolarization (APD_90_) at different BCLs were obtained.

To ensure that the coupled system reached steady state, stimulation was repeated for 20 cycles. The results from the last cycle in each simulation were used. All state variables of the coupled model were updated by means of the forward Euler method. The time step was set to be 10 *μ*s to ensure numerical accuracy and stability.

## 3. Results

### 3.1. *I*_Na_Mfb_ and *I*_MGC_Mfb_ in Mfbs

**Figure 2:**
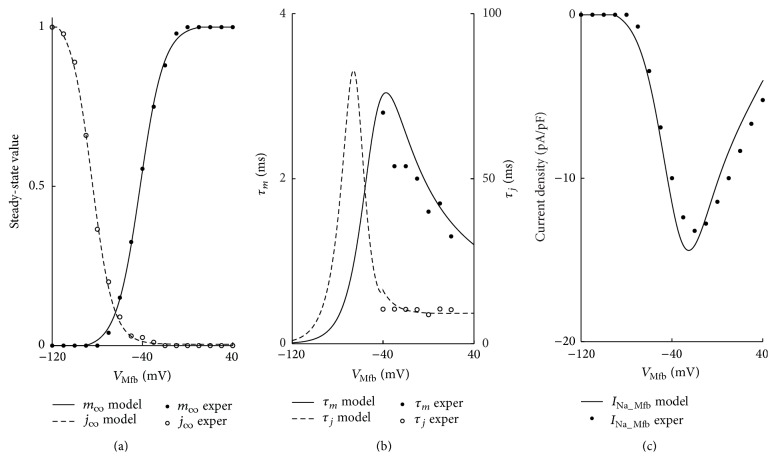
Model representation of parameters describing *I*_Na_Mfb_. (a) Steady-state activation (solid line) and inactivation (dashed line) curves, (b) gating variable fast (solid line) and slow (dashed line) time constants, and (c)* I*-*V* relationship (solid line). Experimental data (filled and hollow circles) from Chatelier et al. is included for comparison.

Figure 2 shows the steady-state activation and inactivation curves, time constants, and the current-voltage (*I*-*V*) relationship of *I*_Na_Mfb_. All the curves were consistent with the experimental data [11].

**Figure 3:**
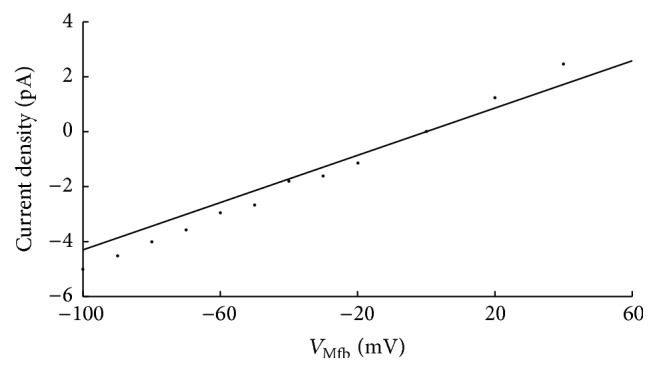
The* I*-*V* relationship (solid line) of MGCs. Experimental data (filled circles) from Kamkin et al. is included for comparison.

Figure 3 shows the linear* I*-*V* relationship of *I*_MGC_Mfb_. The curve was consistent with the experimental data [13].

### 3.2. Effects of *I*_SAC_, *I*_Na_Mfb_, and *I*_MGC_Mfb_ on Atrial Myocyte and Mfbs

**Figure 4:**
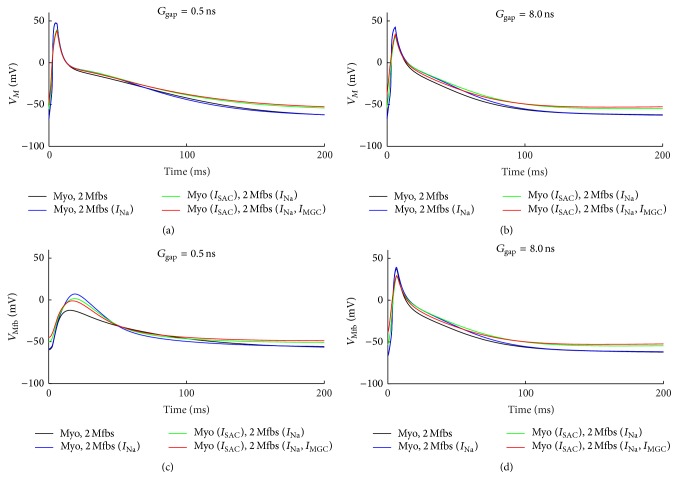
Effects of *I*_SAC_, *I*_Na_Mfb_, and *I*_MGC_Mfb_ on transmembrane potential of myocyte and Mfb. (a, b) *V*_*M*_ and (c, d) *V*_Mfb_, in Mfb-M coupling with *I*_Na_Mfb_ (blue line), with *I*_SAC_ and *I*_Na_Mfb_ (green line), and with *I*_SAC_, *I*_Na_Mfb_, and *I*_MGC_ (red line) as compared to control (black line), by coupling two Mfbs to an atrial myocyte with *G*_gap_ of 0.5 and 8 nS.

Figure 4 shows the combinational effects of *I*_SAC_, *I*_Na_Mfb_, and *I*_MGC_Mfb_ on the membrane potential of myocytes and Mfbs by coupling two Mfbs to a human atrial myocyte with *G*_gap_ of 0.5 and 8 nS. For myocytes, including *I*_Na_Mfb_, *I*_SAC_ and *I*_MGC_Mfb_ to the model of Mfb-M coupling resulted in gradually decreased *V*_max_ and APD_90_ and increased *V*_rest_ depolarization (Figures 4(a) and 4(b)). With *G*_gap_ of 0.5 nS, *V*_max_ was decreased by 0.8% (with *I*_Na_Mfb_), 16.7% (with *I*_Na_Mfb_ and *I*_SAC_), and 18.6% (with *I*_Na_Mfb_, *I*_SAC_, and *I*_MGC_Mfb_) in comparison with the control (without *I*_Na_Mfb_, *I*_SAC_, and *I*_MGC_Mfb_). Correspondingly, for the three conditions, APD_90_ was decreased by 20.0%, 19.1%, and 20.2%, respectively, and *V*_rest_ increased by 8.3%, 20.3%, and 22.4%, respectively. With *G*_gap_ of 8 nS, *V*_max_ was decreased by 0.2%, 23.5%, and 18.6%, respectively. APD_90_ was decreased by 1.6%, 4.1%, and 11.1%, respectively. *V*_rest_ was increased by 0.8%, 12.5%, and 15.9%, respectively. In addition, *I*_Na_Mfb_, *I*_SAC_, and *I*_MGC_Mfb_ decelerated the repolarization of the atrial myocyte AP, especially with a large *G*_gap_. For Mfbs, with *G*_gap_ of 0.5 nS, significant electrotonic depolarizations were observed with the integration of the three currents (Figure 4(c)). *V*_max_ of the Mfb was −12.4 mV (control), 7.1 mV (with *I*_Na_Mfb_), 1.5 mV (with *I*_Na_Mfb_ and *I*_SAC_), and −1.0 mV (with *I*_Na_Mfb_, *I*_SAC_, and *I*_MGC_Mfb_), respectively. With *G*_gap_ of 8 ns, the effects of these currents on Mfbs were similar with that on myocytes (Figure 4(d)).

**Figure 5:**
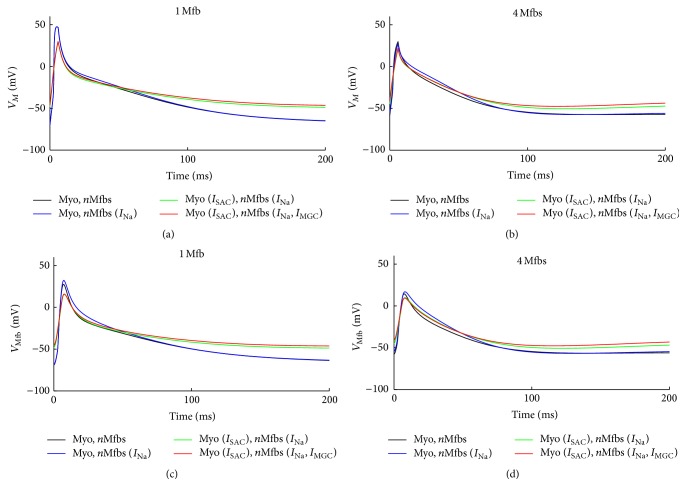
Effects of *I*_SAC_, *I*_Na_Mfb_, and *I*_MGC_Mfb_ on transmembrane potential of myocyte and Mfb. (a, b) *V*_*M*_ and (c, d) *V*_Mfb_, in Mfb-M coupling with *I*_Na_Mfb_ (blue line), with *I*_SAC_ and *I*_Na_Mfb_ (green line), and with *I*_SAC_, *I*_Na_Mfb_ and *I*_MGC_ (red line) as compared to control (black line), by coupling one and four Mfbs to an atrial myocyte with *G*_gap_ of 3 nS.

Figure 5 illustrates similar effects of *I*_SAC_, *I*_Na_Mfb_, and *I*_MGC_Mfb_ on the membrane potential of myocytes and Mfbs when *G*_gap_ was fixed at 3 nS and the number of coupled Mfbs was one or four. For myocytes, integrating these currents into the coupled model resulted in decreased *V*_max_ and APD_90_ and greater depolarization in *V*_rest_ (Figures 5(a) and 5(b)). Coupling one Mfb to a human atrial myocyte resulted in 0.1% (with *I*_Na_Mfb_), 38.1% (with *I*_Na_Mfb_ and *I*_SAC_), and 37.4% (with *I*_Na_Mfb_, *I*_SAC_, and *I*_MGC_Mfb_) decreases in *V*_max_ when compared with the control. Correspondingly, APD_90_ was decreased by 1.1%, 7.7%, and 7.8%, and *V*_rest_ increased by 0.4%, 25.0%, and 28.6%, respectively. Similarly, coupling four Mfbs to a human atrial myocyte resulted in 7.9%, 30.3%, and 25.9% decreases in *V*_max_ when compared with the control. APD_90_ was decreased by 1.6%, 8.9%, and 18.9%, and *V*_rest_ was increased by 2.3%, 17.4%, and 23.6%, respectively. For Mfbs, the effects of these currents were similar with that on myocytes (Figures 5(c) and 5(d)).

**Figure 6:**
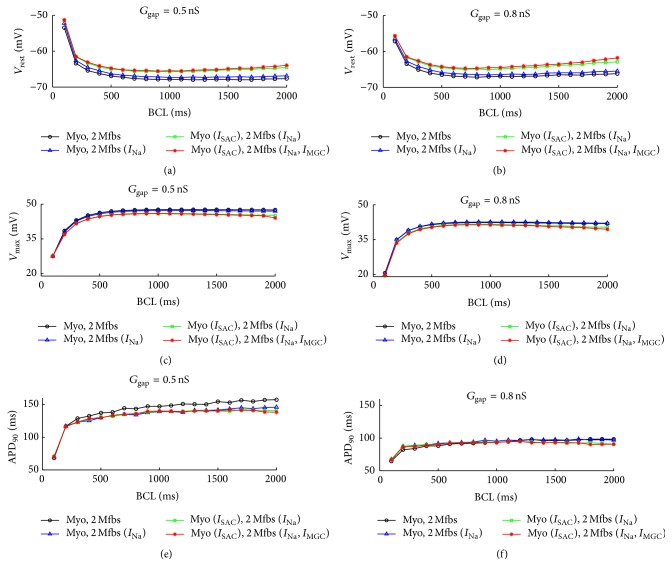
Effects of *I*_SAC_, *I*_Na_Mfb_, and *I*_MGC_Mfb_ on AP characteristics as a function of BCL in Mfb-M coupling with *I*_Na_Mfb_ (blue line with hollow triangle), with *I*_SAC_ and *I*_Na_Mfb_ (green line with hollow rectangle), and with *I*_SAC_, *I*_Na_Mfb_, and *I*_MGC_ (red line with asterisk) as compared to control (black line with hollow circle), by coupling two Mfbs to an atrial myocyte with *G*_gap_ of 0.5 and 8 nS. (a, b) *V*_rest_, (c, d) *V*_max_, and (e, f) APD_90_.

Figure 6 illustrates the changes of  *V*_rest_, *V*_max_, and APD_90_ in myocyte with the integration of *I*_SAC_, *I*_Na_Mfb_, and *I*_MGC_Mfb_ as a function of BCL when coupling two Mfbs to a human atrial myocyte. For both low (0.5 nS, Figures 6(a), 6(c), and 6(e)) and high (8 nS, Figures 6(b), 6(d), and 6(f)) *G*_gap_, *V*_rest_ declined and *V*_max_ rose in the control and with *I*_Na_Mfb_ as BCL increased (Figures 6(a) to 6(d)). *V*_rest_ decreased initially and then increased slightly with *I*_SAC_ and *I*_Na_Mfb_ and with *I*_SAC_, *I*_Na_Mfb_, and *I*_MGC_Mfb_. Contrarily, *V*_max_ increased with BCL initially and then decreased slightly. Gaps of *V*_rest_ and *V*_max_ between the four cases widened as the BCL increased with more pronounced variations for the high *G*_gap_. APD_90_ was prolonged as BCL increased in all the four situations for both *G*_gap_ values (Figures 6(e) and 6(f)). With *G*_gap_ of 0.5 nS, similar to *V*_max_, APD_90_ gradually dropped with *I*_Na_Mfb_, *I*_SAC_, and *I*_MGC_Mfb_ in order at each BCL. However, there was no obvious difference between the four situations with *G*_gap_ of 8 nS.

**Figure 7:**
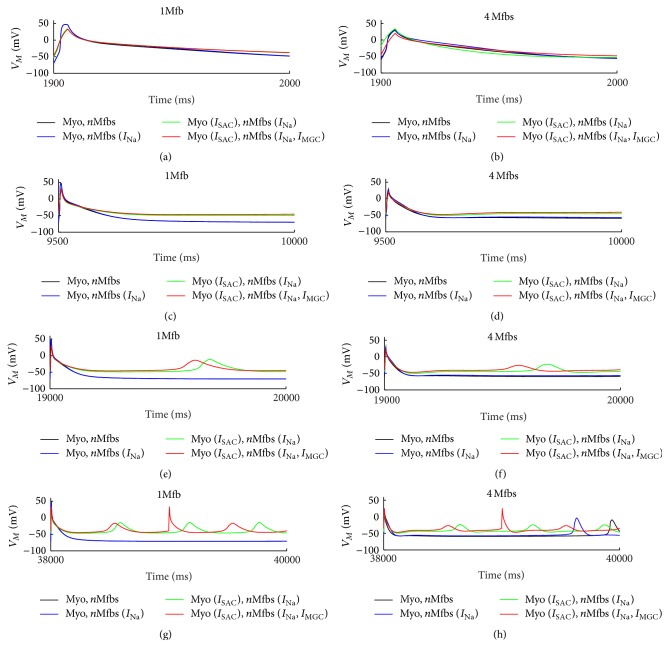
Effects of *I*_SAC_, *I*_Na_Mfb_, and *I*_MGC_Mfb_ on *V*_*M*_ for several BCLs. (a, b) BCL = 100 ms, (c, d) BCL = 500 ms, (e, f) BCL = 1000 ms, and (g, h) BCL = 2000 ms, in Mfb-M coupling with *I*_Na_Mfb_ (blue line), with *I*_SAC_ and *I*_Na_Mfb_ (green line), and with *I*_SAC_, *I*_Na_Mfb_, and *I*_MGC_ (red line) as compared to control (black line), by coupling one and four Mfbs to an atrial myocyte with *G*_gap_ of 3 nS. Results corresponded to the last cycle in each simulation.

Figure 7 shows the APs of the human atrial myocyte at the 20st cycle with BCL of 100 (Figures 7(a) and 7(b)), 500 (Figures 7(c) and 7(d)), 1000 (Figures 7(e) and 7(f)), and 2000 ms (Figures 7(g) and 7(h)) when *G*_gap_ was fixed at 3 nS and the number of coupled Mfbs was one or four. Integrating *I*_SAC_, *I*_Na_Mfb_, and *I*_MGC_Mfb_ into the model of Mfb-M coupling resulted in reductions in *V*_max_ and APD_90_ and greater depolarization in *V*_rest_. However, it is noted that spontaneous excitements in the myocyte appeared when the value of BCL was higher than a certain value (1000 ms in this study). The increased *V*_rest_ and the longer stimulus interval were the two major factors for the spontaneous excitement. Meanwhile, integrating *I*_SAC_, *I*_Na_Mfb_, and *I*_MGC_Mfb_ simultaneously into the coupled model triggered the most and earliest spontaneous excitement at BCL of 1000 and 2000 ms, suggesting that the three currents could result in discordant alternans, especially with large BCL (2000 ms in this study).

## 4. Discussion

This study investigated the roles of *I*_SAC_, *I*_Na_Mfb_, and *I*_MGC_Mfb_ in the excitability and AP. To address these issues, numerical simulations of the coupled Mfb-M system were performed by employing a combination of models of the human atrial myocyte (including *I*_SAC_) and Mfb, as well as modified formulation of *I*_Na_Mfb_ and *I*_MGC_Mfb_ based on experimental data. Specifically, the effects of these currents with changes in (1) *G*_gap_, (2) the number of Mfbs, and (3) BCL on the human atrial myocyte AP dynamics were investigated. The main results with the integration of *I*_SAC_, *I*_Na_Mfb_, and *I*_MGC_Mfb_ were as follows: (1) for myocytes, the addition of *I*_SAC_, *I*_Na_Mfb_, and *I*_MGC_Mfb_ resulted in decreased *V*_max_ and APD_90_ and increased *V*_rest_ depolarization; deceleration of AP repolarization; and trigger of spontaneous excitements even discordant alternans at large BCLs and (2) for Mfbs, significant electrotonic depolarizations were obtained with small *G*_gap_. As the value of *G*_gap_ and the number of coupled Mfbs increased, the effects for Mfbs were similar to those in myocytes.

### 4.1. Resting Potential of Human Atrial Mfbs

*V*_rest_ of fibroblasts/Mfbs in human atria was close to −15 mV [33, 34]. At this potential, the persistent entry of Na^+^ in Mfb would be negligible [11]. Indeed, it has been reported that *V*_rest_ of these cells was sensitive to mechanical stress under both contraction and relaxation [13, 35, 36]. A stretch of 3 *μ*m led to a negative shift of *V*_rest_, about −35 ± 5 mV [13]. Reoxygenation after hypoxia also produced a hyperpolarization to *V*_rest_ [37]. Based on these findings, we set up the mathematical formulation of *I*_MGC_Mfb_ and included it in the Mfb-M coupling model. In addition, it has also been shown that K^+^ current in cardiac fibroblasts and *V*_rest_ was between −40.0 and −60.0 mV [8, 9, 38]. These potentials corresponded to the peak of the Na^+^ window current described by Chatelier et al. [11]. A persistent Na^+^ entry may be induced based on such a cell polarity. Therefore, we also introduced the mathematical formulation of *I*_Na_Mfb_ and included it in Mfb modeling. *V*_rest_ was set to be −47.8 mV, within the range of experimental findings [8, 9].

### 4.2. Role of *I*_SAC_, *I*_Na_Mfb_, and *I*_MGC_Mfb_ on *V*_rest_ and Excitability of Human Atrial Myocytes

Both experimental data and computational work have shown that the value of *G*_gap_ and the number of coupled fibroblasts/Mfbs were important in determining the depolarization of the coupled human myocyte at rest [24, 28, 29, 39]. Based on the preceding conclusions, our simulations also considered the effects of *I*_SAC_, *I*_Na_Mfb_, and *I*_MGC_Mfb_ on *V*_rest_ and excitability of human atrial myocytes.

For *I*_SAC_, previous studies explored the electrophysiological effect of sustained stretch and indicated that *I*_SAC_ influenced cardiac myocytes repolarization and activation [26, 40]. With pathophysiological conditions, such as hypertension and AF, it has been observed that atrial stretch and dilatation influenced atrial flutter cycle length and facilitated the induction and maintenance of AF [41, 42]. However, the SAC blocker Gd^3+^ reduced the stretch-induced vulnerability to AF [43]. These observational studies confirmed that *I*_SAC_ plays a significant role in heart pathology. In our simulations, integrating *I*_SAC_ into the mathematical model of the human atrial myocyte resulted in a significant depolarization of *V*_rest_ and prolongation of repolarization (Figures 4 to 7). These phenomena led to alternating spontaneous excitement propagation at a BCL of 2000 ms (Figure 7). Our simulation agreed with Kuijpers et al., where slow conduction, a longer effective refractory period, and unidirectional conduction block with increasing stretch were observed [26], which were related to the inducibility of AF.

For *I*_Na_Mfb_, many studies have been conducted to investigate how this current could influence Mfb proliferation, since fibrotic remodeling was closely related to AF. It has been reported that Na_v_ 1.5 *α*-subunit was responsible of *I*_Na_Mfb_ in human atrial Mfbs, which represented electrophysiological characteristics similar to Na^+^ channels found in cardiac excitable cells [11, 44]. Based on this, a multiple parameter exponential function was used in this study, which was modeled after the equation of time courses by Courtemanche et al. to fit the time courses of current decay elicited at depolarized voltages [31]. Unlike the assumption from Koivumäki et al. that *V*_rest_ of Mfbs resulted in significant inactivation of *I*_Na_Mfb_ [45], the current was activated during an atrial Mfb AP in our simulations. Our results showed that *I*_Na_Mfb_ decreased *V*_max_ and APD_90_ and increased *V*_rest_ depolarization in myocytes (Figures 4 to 7). Like *I*_SAC_, this depolarization would be expected to change diastolic Ca^2+^ levels and conduction velocity.

For *I*_MGC_Mfb_, experimental data has indicated that cardiac fibroblasts expressed functional MCGs, contributing to the cardiac mechanoelectrical feedback under both physiological and pathophysiological conditions [18, 19]. Since *I*_MGC_Mfb_ has been found in Mfbs, we assumed that it could affect Mfb electrophysiological characteristics like *I*_SAC_ on myocyte. In our simulations, the* I*-*V* relation for single MGCs was linear, which agreed with the experimental results [13]. The results showed that integrating *I*_MGC_Mfb_ in the Mfb model changed the membrane potential of Mfb and further influenced the membrane potential of myocyte by Mfb-myocyte coupling (Figures 4 to 7). Interestingly, MGCs were activated by Mfb compression and inactivated by Mfb stretch [13], implying that *I*_MGC_Mfb_ should be integrated in cell modeling only during cell compression, such as fibroblasts/Mfbs compression caused by stretching and dilatation of surrounding cardiac myocytes.

Table 1 shows changes in myocyte *V*_rest_, *V*_max_, and APD_90_ in three situations as compared to control. The situations were Mfb-M coupling with (1) *I*_Na_Mfb_, (2) *I*_Na_Mfb_ and *I*_SAC_, and (3) *I*_Na_Mfb_, *I*_SAC_, and *I*_MGC_Mfb_, respectively. Values were recorded with two Mfbs coupled to a single atrial myocyte in which *G*_gap_ was varied between 0.5 and 8 nS, or with one and four identical Mfbs coupled to a single atrial myocyte at a constant *G*_gap_ of 3 nS. In general, it can be seen that *I*_Na_Mfb_, *I*_SAC_, and *I*_MGC_Mfb_ played a greater role in reducing *V*_max_ and APD_90_ and increasing the depolarization of *V*_rest_ when *G*_gap_ was relatively small. Increasing the number of coupled Mfbs contributed to a further decline in APD_90_ in all cases, while having different influences on *V*_max_ and *V*_rest_. The reduction of *V*_max_ and the rise of depolarization of *V*_rest_ were increased in case (1) and both declined in case (2) and (3).

Previous modeling work suggested that Mfb-M coupling contributed to arrhythmia formation [23, 46]. The key factors included *G*_gap_, the number of coupled Mfbs, and Mfb density. Here, we investigated the functional effect of fibrotic remodeling on AF by integrating *I*_Na_Mfb_, *I*_SAC_, and *I*_MGC_Mfb_ into Mfb-M coupling. To the best of our knowledge, this has not been examined before. As summarized in Figure 7, these currents made the Mfb act as a current source for the cardiac myocyte, leading to depolarization of *V*_rest_ and shortening of APD, thereby preserving myocyte excitability to trigger spontaneous excitement and facilitating reentry (such as those of AF).

### 4.3. Limitations

Two limitations of this mathematical modeling should be mentioned. First, the influence of homologous coupling between adjacent Mfbs was not considered. Since Mfbs can form conduction bridges between myocyte bundles and introduce nonlinearity into Mfb-M coupling to alter electrophysiological properties of the coupled myocyte, coupling between Mfbs may be important in tissue modeling [28, 29]. Second, our modeling of *I*_MGC_Mfb_ was done using a simplified model based on a linear equation. We did not include the mechanosensitivity of MGCs in our simulation; for example, the conductance depends of the mechanical forces to which the cells are submitted.

## 5. Conclusions

This study demonstrated the combinational effects of *I*_Na_Mfb_ and *I*_MGC_Mfb_ in Mfbs on myocyte excitability and repolarization. Our results showed that the addition of *I*_Na_Mfb_ and *I*_MGC_Mfb_ reduced *V*_max_, shortened APD, depolarized *V*_rest_, and was easy to trigger spontaneous excitement in myocytes. The effects proved that Na^+^ current and mechanogated channels in Mfbs should be considered in future pathological cardiac mathematical modeling, such as atrial fibrillation and cardiac fibrosis.

## Supplementary Material

The Supplementary Material contained model equations, parameters values and initial conditions necessary to carry out the simulations presented in this article. The model equations included equations of the atrial myocyte model (Table 1 through 12), the atrial myofibroblast model (Table 13 through 19) and myofibroblast-myocyte coupling (Table 20). Parameters values and initial conditions were given in Table 21 through 23. Glossary was listed at the end.

## Figures and Tables

**Table 1:** Percentage changes in myocyte *V*_rest_, *V*_max_ and APD_90_ as compared to control.

		*G*_gap_ (nS)		Coupled Mfbs	
		0.5	8	1	4
*V*_max_↓ (%)	*I*_Na_Mfb_	0.8	0.2	0.1	7.9
	*I*_Na_Mfb_, *I*_SAC_	16.7	23.5	38.1	30.3
	*I*_Na_Mfb_, *I*_SAC_, *I*_MGC_Mfb_	18.6	18.6	37.4	25.9
APD↓ (%)	*I*_Na_Mfb_	20.0	1.6	1.1	1.6
	*I*_Na_Mfb_, *I*_SAC_	19.1	4.1	7.7	8.9
	*I*_Na_Mfb_, *I*_SAC_, *I*_MGC_Mfb_	20.2	11.1	7.8	18.9
*V*_rest_↑ (%)	*I*_Na_Mfb_	8.3	0.8	0.4	2.3
	*I*_Na_Mfb_, *I*_SAC_	20.3	12.5	25.0	17.4
	*I*_Na_Mfb_, *I*_SAC_, *I*_MGC_Mfb_	22.4	15.9	28.6	23.6
