# Supplementary material for: Effects of Na+ Current and Mechanogated Channels in Myofibroblasts on Myocyte Excitability and Repolarization
Source: Comput Math Methods Med. 2016 Nov 17;2016:6189374. doi: 10.1155/2016/6189374 (PMC5131562; doi:10.1155/2016/6189374)
Supplement: Supplementary file 1 — The Supplementary Material contained model equations, parameters values and initial conditions necessary to carry out the simulations presented in this article. The model equations included equations of the atrial myocyte model (Table 1 through 12), the atrial myofibroblast model (Table 13 through 19) and myofibroblast-myocyte coupling (Table 20). Parameters values and initial conditions were given in Table 21 through 23. Glossary was listed at the end. [file 6189374.f1.pdf]

Supplementary Material

Model Equations

Table 1 through 23 contained all the equations, parameters values and initial conditions necessary to carry out the simulations presented in this article. Unless otherwise noted, the units are as follows: time in seconds (s), voltage in millivolts (mV), concentration in millimoles/liter (mmol/L), current in picoamperes (pA), conductance in nanosiemens (nS), capacitance in picofarads (pF), volume in nanoliters (nL), and temperature in kelvin (K).

Atrial Myocyte Model

The atrial myocyte model was represented by the Maleckar et al. mathematical model [1], which was based on a previous model of the adult human atrial myocyte action potential (AP), that of Nygren et al. [2]. The stimulus used to evoke an AP was a rectangular current pulse (I_{stim}) with amplitude of 280 pA and duration of 6 ms. Equations of the stretch-activated current (I_{SAC}) were taken from Kuijpers et al [3], which were assumed to be a nonselective cation current with a near-linear current-voltage relation on the basis of experimental observations [4]. When I_{SAC} was integrated in the myofibroblast-myocyte (Mfb-M) coupling, equations of intracellular ion concentrations of Na^+, K^+ and Ca^{2+} ($[Na^+]_i$, $[K^+]_i$, and $[Ca^{2+}]_i$) followed Table 12. Without I_{SAC} in Mfb-M coupling, they followed Table 8.

Table 1. Na^+ current: I_{Na}

$$I_{\text{Na}} = P_{\text{Na}} m^3 (0.9h_1 + 0.1h_2)[Na^+]_c V_{\text{M}} \frac{\text{F}^2}{\text{RT}} \frac{e^{(V_{\text{M}}-E_{\text{Na}})\text{F}/\text{RT}} - 1.0}{e^{V_{\text{M}}\text{F}/\text{RT}} - 1.0}$$

$$\bar{m} = \frac{1.0}{1.0 + e^{(V_{\text{M}}+27.12)/-8.21}}$$

$$\bar{h} = \frac{1.0}{1.0 + e^{(V_{\text{M}}+63.6)/5.3}}$$

$$\frac{dm}{dt} = \frac{\bar{m} - m}{\tau_m}$$

$$\tau_m = 0.000042e^{-((V_{\text{M}}+25.57)/28.8)^2} + 0.000024$$

$$\frac{dh_1}{dt} = \frac{\bar{h} - h_1}{\tau_{h_1}}$$

$$\tau_{h_1} = \frac{0.03}{1.0 + e^{(V_{\text{M}}+35.1)/3.2}} + 0.0003$$

$$\frac{dh_2}{dt} = \frac{\bar{h} - h_2}{\tau_{h_2}}$$

$$\tau_{h_2} = \frac{0.12}{1.0 + e^{(V_{\text{M}}+35.1)/3.2}} + 0.003$$

$$E_{\text{Na}} = \frac{\text{RT}}{\text{F}} \log \frac{[\text{Na}^+]_c}{[\text{Na}^+]_i}$$

Table 2. Ca^{2+} current: I_{CaL}

$$I_{\text{CaL}} = \bar{g}_{\text{CaL}} d_{\text{L}} [f_{\text{Ca}} f_{\text{L1}} + (1 - f_{\text{Ca}}) f_{\text{L2}}](V_{\text{M}} - E_{\text{Ca,app}})$$

$$\bar{d}_L = \frac{1.0}{1.0 + e^{(V_M+9.0)/\text{-}5.8}}$$

$$\bar{f}_L = \frac{1.0}{1.0 + e^{(V_M+27.4)/7.1}}$$

$$\frac{dd_L}{dt} = \frac{\bar{d}_L - d_L}{\tau_{d_L}}$$

$$\tau_{d_L} = 0.0027e^{-((V_M+35.0)/30.0)^2} + 0.002$$

$$\frac{df_{L1}}{dt} = \frac{\bar{f}_L - f_{L1}}{\tau_{f_{L1}}}$$

$$\tau_{f_{L1}} = 0.161e^{-((V_M+40.0)/14.4)^2} + 0.01$$

$$\frac{df_{L2}}{dt} = \frac{\bar{f}_L - f_{L2}}{\tau_{f_{L2}}}$$

$$\tau_{f_{L2}} = 1.3323e^{-((V_M+40.0)/14.2)^2} + 0.0626$$

$$f_{Ca} = \frac{[Ca^{2+}]_d}{[Ca^{2+}]_d + k_{Ca}}$$

Table 3. Transient and ultrarapidly delayed rectifier K^+ currents: I_t and I_{Kur}

$$I_t = \bar{g}_t r s (V_M - E_K)$$

$$\bar{r} = \frac{1.0}{1.0 + e^{(V_M-1.0)/\text{-}11.0}}$$

$$\bar{s} = \frac{1.0}{1.0 + e^{(V_M+40.5)/11.5}}$$

$$\frac{dr}{dt} = \frac{\bar{r} - r}{\tau_r}$$

$$\tau_r = 0.0035e^{-(V_M/30.0)^2} + 0.0015$$

$$\frac{ds}{dt} = \frac{\bar{s} - s}{\tau_s}$$

$$\tau_s = 0.025635e^{-((V_M+52.45)/15.89)^2} + 0.01414$$

$$I_{Kur} = \bar{g}_{Kur} r_{Kur} s_{Kur} (V_M - E_K)$$

$$\bar{r}_{Kur} = \frac{1.0}{1.0 + e^{(V_M+6.0)/\text{-}8.6}}$$

$$\bar{s}_{Kur} = \frac{1.0}{1.0 + e^{(V_M+7.5)/10.0}}$$

$$\frac{dr_{Kur}}{dt} = \frac{\bar{r}_{Kur} - r_{Kur}}{\tau_{r_{Kur}}}$$

$$\tau_{r_{Kur}} = \frac{0.009}{1.0 + e^{(V_M+5.0)/12.0}} + 0.0005$$

$$\frac{ds_{Kur}}{dt} = \frac{\bar{s}_{Kur} - s_{Kur}}{\tau_{s_{Kur}}}$$

$$\tau_{s_{Kur}} = \frac{0.59}{1.0 + e^{(V_M+60.0)/10.0}} + 3.05$$

$$E_K = \frac{RT}{F}\log\frac{[K^+]_c}{[K^+]_i}$$

Table 4. Delayed rectifier K^+ currents: $I_{K,s}$ and $I_{K,r}$

$$I_{K,s} = \bar{g}_{K,s} n (V_M - E_K)$$

$$\bar{n} = \frac{1.0}{1.0 + e^{(V_M-19.9)/\text{-}12.7}}$$

$$\tau_n = 0.7 + 0.4e^{-((V_M-20.0)/20.0)^2}$$

$$\frac{dn}{dt} = \frac{\bar{n} - n}{\tau_n}$$

$$E_K = \frac{RT}{F}\log\frac{[K^+]_c}{[K^+]_i}$$

$$I_{K,r} = \bar{g}_{K,r} p_a p_i (V_M - E_K)$$

$$\bar{p}_a = \frac{1.0}{1.0 + e^{(V_M+15.0)/\text{-}6.0}}$$

$$p_i = \frac{1.0}{1.0 + e^{(V_M+55.0)/24.0}}$$

$$\frac{dp_a}{dt} = \frac{\bar{p}_a - p_a}{\tau_{p_a}}$$

$$\tau_{p_a} = 0.03118 + 0.21718e^{-((V_M+20.1376)/22.1996)^2}$$

Table 5. Inward rectifier K$^+$ currents: I_{K1}

$$I_{\mathrm{K1}} = \bar{g}_{\mathrm{K1}} [\mathrm{K}^+]_{\mathrm{c}}^{0.4457} \frac{V_{\mathrm{M}} - E_{\mathrm{K}}}{1.0 + e^{1.5(V_{\mathrm{M}} - E_{\mathrm{K}} + 3.6)\mathrm{F/RT}}}$$

$$E_{\mathrm{K}} = \frac{\mathrm{RT}}{\mathrm{F}} \log \frac{[\mathrm{K}^+]_{\mathrm{c}}}{[\mathrm{K}^+]_{\mathrm{i}}}$$

Table 6. Background inward currents: $I_{B,Na}$ and $I_{B,Ca}$

$$I_{\mathrm{B,Na}} = \bar{g}_{\mathrm{B,Na}} (V_{\mathrm{M}} - E_{\mathrm{Na}}) \qquad I_{\mathrm{B,Ca}} = \bar{g}_{\mathrm{B,Ca}} (V_{\mathrm{M}} - E_{\mathrm{Ca}})$$

$$E_{\mathrm{Na}} = \frac{\mathrm{RT}}{\mathrm{F}} \log \frac{[\mathrm{Na}^+]_{\mathrm{c}}}{[\mathrm{Na}^+]_{\mathrm{i}}} \qquad E_{\mathrm{Ca}} = \frac{\mathrm{RT}}{2\mathrm{F}} \log \frac{[\mathrm{Ca}^{2+}]_{\mathrm{c}}}{[\mathrm{Ca}^{2+}]_{\mathrm{i}}}$$

Table 7. Pump and exchanger currents: I_{NaK}, I_{CaP}, and I_{NaCa}

$$I_{\mathrm{NaK}} = \bar{I}_{\mathrm{NaK}} \frac{[\mathrm{K}^+]_{\mathrm{c}}}{[\mathrm{K}^+]_{\mathrm{c}} + k_{\mathrm{NaK,K}}} \cdot \frac{[\mathrm{Na}^+]_{\mathrm{i}}^{1.5}}{[\mathrm{Na}^+]_{\mathrm{i}}^{1.5} + k_{\mathrm{NaK,Na}}^{1.5}} \cdot \frac{V_{\mathrm{M}} + 150.0}{V_{\mathrm{M}} + 200.0}$$

$$I_{\mathrm{CaP}} = \bar{I}_{\mathrm{CaP}} \frac{[\mathrm{Ca}^{2+}]_{\mathrm{i}}}{[\mathrm{Ca}^{2+}]_{\mathrm{i}} + k_{\mathrm{CaP}}}$$

$$I_{\mathrm{NaCa}} = k_{\mathrm{NaCa}} \frac{[\mathrm{Na}^+]_{\mathrm{i}}^3 [\mathrm{Ca}^{2+}]_{\mathrm{c}} e^{\gamma \mathrm{VF/RT}} - [\mathrm{Na}^+]_{\mathrm{c}}^3 [\mathrm{Ca}^{2+}]_{\mathrm{i}} e^{(\gamma - 1.0)\mathrm{VF/RT}}}{1.0 + d_{\mathrm{NaCa}} \left([\mathrm{Na}^+]_{\mathrm{c}}^3 [\mathrm{Ca}^{2+}]_{\mathrm{i}} + [\mathrm{Na}^+]_{\mathrm{i}}^3 [\mathrm{Ca}^{2+}]_{\mathrm{c}} \right)}$$

Table 8. Intracellular ion concentrations: $[Na^+]_i$, $[K^+]_i$, and $[Ca^{2+}]_i$

$$\frac{d[\mathrm{Na}^+]_{\mathrm{i}}}{dt} = -\frac{I_{\mathrm{Na}} + I_{\mathrm{B,Na}} + 3I_{\mathrm{NaK}} + 3I_{\mathrm{NaCa}}}{\mathrm{Vol_i F}}$$

$$\frac{d[\mathrm{K}^+]_{\mathrm{i}}}{dt} = -\frac{I_{\mathrm{t}} + I_{\mathrm{Kur}} + I_{\mathrm{K1}} + I_{\mathrm{K,s}} + I_{\mathrm{K,r}} - 2I_{\mathrm{NaK}}}{\mathrm{Vol_i F}}$$

$$\frac{d[\mathrm{Ca}^{2+}]_{\mathrm{i}}}{dt} = -\frac{-I_{\mathrm{di}} + I_{\mathrm{B,Ca}} + I_{\mathrm{CaP}} - 2I_{\mathrm{NaCa}} + I_{\mathrm{up}} - I_{\mathrm{rel}}}{2.0\ \mathrm{Vol_i F}} - \frac{dO}{dt}$$

$$\frac{dO}{dt} = 0.08 \frac{dO_{\mathrm{TC}}}{dt} + 0.16 \frac{dO_{\mathrm{TMgC}}}{dt} + 0.045 \frac{dO_{\mathrm{C}}}{dt}$$

$$\frac{d[\mathrm{Ca}^{2+}]_{\mathrm{d}}}{dt} = -\frac{I_{\mathrm{CaL}} - I_{\mathrm{di}}}{2.0\ \mathrm{Vol_d F}}$$

$$I_{\mathrm{di}} = ([\mathrm{Ca}^{2+}]_{\mathrm{d}} - [\mathrm{Ca}^{2+}]_{\mathrm{i}}) \frac{2\mathrm{F\ Vol_d}}{\tau_{\mathrm{di}}}$$

Table 9. Cleft space ion concentrations: $[Na^+]_c$, $[K^+]_c$, and $[Ca^{2+}]_c$

$$\frac{d[\mathrm{Na}^+]_{\mathrm{c}}}{dt} = \frac{[\mathrm{Na}^+]_{\mathrm{b}} - [\mathrm{Na}^+]_{\mathrm{c}}}{\tau_{\mathrm{Na}}} + \frac{I_{\mathrm{Na}} + I_{\mathrm{B,Na}} + 3I_{\mathrm{NaK}} + 3I_{\mathrm{NaCa}}}{\mathrm{Vol_c F}}$$

$$\frac{d[\mathrm{K}^+]_{\mathrm{c}}}{dt} = \frac{[\mathrm{K}^+]_{\mathrm{b}} - [\mathrm{K}^+]_{\mathrm{c}}}{\tau_{\mathrm{K}}} + \frac{I_{\mathrm{t}} + I_{\mathrm{Kur}} + I_{\mathrm{K1}} + I_{\mathrm{K,s}} + I_{\mathrm{K,r}} - 2I_{\mathrm{NaK}}}{\mathrm{Vol_c F}}$$

$$\frac{d[\mathrm{Ca}^{2+}]_{\mathrm{c}}}{dt} = \frac{[\mathrm{Ca}^{2+}]_{\mathrm{b}} - [\mathrm{Ca}^{2+}]_{\mathrm{c}}}{\tau_{\mathrm{Ca}}} + \frac{I_{\mathrm{CaL}} + I_{\mathrm{B,Ca}} + I_{\mathrm{CaP}} - 2I_{\mathrm{NaCa}}}{2.0\ \mathrm{Vol_c F}}$$

Table 10. Intracellular Ca^{2+} buffering

$$\frac{dO_{\mathrm{C}}}{dt} = 200000.0[\mathrm{Ca}^{2+}]_{\mathrm{i}}(1.0 - O_{\mathrm{C}}) - 476.0O_{\mathrm{C}}$$

$$\frac{dO_{\mathrm{TC}}}{dt} = 78400.0[\mathrm{Ca}^{2+}]_{\mathrm{i}}(1.0 - O_{\mathrm{TC}}) - 392.0O_{\mathrm{TC}}$$

$$\frac{dO_{\mathrm{TMgC}}}{dt} = 200000.0[\mathrm{Ca}^{2+}]_{\mathrm{i}}\left(1.0 - O_{\mathrm{TMgC}} - O_{\mathrm{TMgMg}}\right) - 6.6O_{\mathrm{TMgC}}$$

$$\frac{dO_{\mathrm{TMgMg}}}{dt} = 2000.0[\mathrm{Mg}^{2+}]_{\mathrm{i}}\left(1.0 - O_{\mathrm{TMgC}} - O_{\mathrm{TMgMg}}\right) - 666.0O_{\mathrm{TMgMg}}$$

Table 11. Ca^{2+} handling by the sarcoplasmic reticulum

$$I_{\mathrm{up}} = \bar{I}_{\mathrm{up}}\frac{[\mathrm{Ca}^{2+}]_{\mathrm{i}}/k_{\mathrm{cyca}} - k_{\mathrm{xcs}}^{2}[\mathrm{Ca}^{2+}]_{\mathrm{up}}/k_{\mathrm{srca}}}{\left([\mathrm{Ca}^{2+}]_{\mathrm{i}} + k_{\mathrm{cyca}}\right)/k_{\mathrm{cyca}} + k_{\mathrm{xcs}}\left([\mathrm{Ca}^{2+}]_{\mathrm{up}} + k_{\mathrm{srca}}\right)/k_{\mathrm{srca}}}$$

$$I_{\mathrm{tr}} = \left([\mathrm{Ca}^{2+}]_{\mathrm{up}} - [\mathrm{Ca}^{2+}]_{\mathrm{rel}}\right)\frac{2\mathrm{F\,Vol}_{\mathrm{rel}}}{\tau_{\mathrm{tr}}}$$

$$I_{\mathrm{rel}} = \alpha_{\mathrm{rel}}\left(\frac{F_2}{F_2 + 0.25}\right)^2 \left([\mathrm{Ca}^{2+}]_{\mathrm{rel}} - [\mathrm{Ca}^{2+}]_{\mathrm{i}}\right)$$

$$\frac{dO_{\mathrm{Calse}}}{dt} = 480.0[\mathrm{Ca}^{2+}]_{\mathrm{rel}}(1.0 - O_{\mathrm{Calse}}) - 400.0O_{\mathrm{Calse}}$$

$$\frac{d[\mathrm{Ca}^{2+}]_{\mathrm{rel}}}{dt} = \frac{I_{\mathrm{tr}} - I_{\mathrm{rel}}}{2\mathrm{F\,Vol}_{\mathrm{rel}}} - 31.0\frac{dO_{\mathrm{Calse}}}{dt}$$

$$\frac{d[\mathrm{Ca}^{2+}]_{\mathrm{up}}}{dt} = \frac{I_{\mathrm{up}} - I_{\mathrm{tr}}}{2\mathrm{F\,Vol}_{\mathrm{up}}}$$

$$\frac{dF_1}{dt} = \mathrm{r}_{\mathrm{recov}}(1.0 - F_1 - F_2) - r_{\mathrm{act}}F_1$$

$$\frac{dF_2}{dt} = r_{\mathrm{act}}F_1 - r_{\mathrm{inact}}F_2$$

$$r_{\mathrm{act}} = 203.8\left[\left(\frac{[\mathrm{Ca}^{2+}]_{\mathrm{i}}}{[\mathrm{Ca}^{2+}]_{\mathrm{i}} + k_{\mathrm{rel,i}}}\right)^4 + \left(\frac{[\mathrm{Ca}^{2+}]_{\mathrm{d}}}{[\mathrm{Ca}^{2+}]_{\mathrm{d}} + k_{\mathrm{rel,d}}}\right)^4\right]$$

$$r_{\mathrm{inact}} = 33.96 + 339.6\left(\frac{[\mathrm{Ca}^{2+}]_{\mathrm{i}}}{[\mathrm{Ca}^{2+}]_{\mathrm{i}} + k_{\mathrm{rel,i}}}\right)^4$$

Table 12. Stretch-activated current: I_{SAC}

$$I_{\mathrm{SAC}} = I_{\mathrm{SAC,Na}} + I_{\mathrm{SAC,K}} + I_{\mathrm{SAC,Ca}}$$

$$I_{\mathrm{SAC,Na}} = \bar{P}_{\mathrm{Na}}g_{\mathrm{SAC}}\frac{\mathrm{z}_{\mathrm{Na}}^2\mathrm{F}^2V_{\mathrm{M}}}{\mathrm{RT}}\cdot\frac{[\mathrm{Na}^+]_{\mathrm{i}} - [\mathrm{Na}^+]_{\mathrm{c}}e^{-(z_{\mathrm{Na}}\mathrm{F}V_{\mathrm{M}})/\mathrm{RT}}}{1.0 - e^{-(z_{\mathrm{Na}}\mathrm{F}V_{\mathrm{M}})/\mathrm{RT}}}$$

$$I_{\mathrm{SAC,K}} = \bar{P}_{\mathrm{K}}g_{\mathrm{SAC}}\frac{\mathrm{z}_{\mathrm{K}}^2\mathrm{F}^2V_{\mathrm{M}}}{\mathrm{RT}}\cdot\frac{[\mathrm{K}^+]_{\mathrm{i}} - [\mathrm{K}^+]_{\mathrm{c}}e^{-(z_{\mathrm{K}}\mathrm{F}V_{\mathrm{M}})/\mathrm{RT}}}{1.0 - e^{-(z_{\mathrm{K}}\mathrm{F}V_{\mathrm{M}})/\mathrm{RT}}}$$

$$I_{\mathrm{SAC,Ca}} = \bar{P}_{\mathrm{Ca}}g_{\mathrm{SAC}}\frac{\mathrm{z}_{\mathrm{Ca}}^2\mathrm{F}^2V_{\mathrm{M}}}{\mathrm{RT}}\cdot\frac{[\mathrm{Ca}^{2+}]_{\mathrm{i}} - [\mathrm{Ca}^{2+}]_{\mathrm{c}}e^{-(z_{\mathrm{Ca}}\mathrm{F}V_{\mathrm{M}})/\mathrm{RT}}}{1.0 - e^{-(z_{\mathrm{Ca}}\mathrm{F}V_{\mathrm{M}})/\mathrm{RT}}}$$

$$g_{\mathrm{SAC}} = \frac{G_{\mathrm{SAC}}}{1.0 + K_{\mathrm{SAC}} e^{-(\alpha_{\mathrm{SAC}}(\lambda-1))}}$$

$$\bar{P}_{\mathrm{Na}}:\bar{P}_{\mathrm{K}}:\bar{P}_{\mathrm{Ca}} = 1:1:1$$

$$\frac{d[\mathrm{Na}^+]_{\mathrm{i}}}{dt} = -\frac{I_{\mathrm{Na}} + I_{\mathrm{B,Na}} + 3I_{\mathrm{NaK}} + 3I_{\mathrm{NaCa}} + I_{\mathrm{SAC,Na}}}{\mathrm{Vol_i F}}$$

$$\frac{d[\mathrm{K}^+]_{\mathrm{i}}}{dt} = -\frac{I_{\mathrm{t}} + I_{\mathrm{Kur}} + I_{\mathrm{K1}} + I_{\mathrm{K,s}} + I_{\mathrm{K,r}} - 2I_{\mathrm{NaK}} + I_{\mathrm{SAC,K}}}{\mathrm{Vol_i F}}$$

$$\frac{d[\mathrm{Ca}^{2+}]_{\mathrm{i}}}{dt} = -\frac{-I_{\mathrm{di}} + I_{\mathrm{B,Ca}} + I_{\mathrm{CaP}} - 2I_{\mathrm{NaCa}} + I_{\mathrm{up}} - I_{\mathrm{rel}} + I_{\mathrm{SAC,Ca}}}{2.0\ \mathrm{Vol_i F}} - \frac{dO}{dt}$$

Atrial Myofibroblast Model

The atrial Mfb model was represented by the MacCannell et al. mathematical model [5]. Mathematical formulations of the currents through voltage-gated sodium channels ($I_{\mathrm{Na\_Mfb}}$) and mechano-gated channels ($I_{\mathrm{MGC\_Mfb}}$) was based on experimental data from Chatelier et al. [6] and Kamkin et al. [7], respectively.

Table 13. Time- and voltage-dependent K$^+$ current: I$_{\mathrm{Kv_Mfb}}$

$$I_{\mathrm{Kv\_Mfb}} = \bar{g}_{\mathrm{Kv,Mfb}} r_{\mathrm{Kv}} s_{\mathrm{Kv}} \left(V_{\mathrm{Mfb}} - E_{\mathrm{K,Mfb}}\right)$$

$$\bar{r}_{\mathrm{Kv}} = \left(1.0 + e^{-(V_{\mathrm{Mfb}}+20.0)/11.0}\right)^{-1}$$

$$\bar{s}_{\mathrm{Kv}} = \left(1.0 + e^{(V_{\mathrm{Mfb}}+23.0)/7.0}\right)^{-1}$$

$$\frac{dr_{\mathrm{Kv}}}{dt} = \frac{\bar{r}_{\mathrm{Kv}} - r_{\mathrm{Kv}}}{\tau_{r_{\mathrm{Kv}}}}$$

$$\tau_{r_{\mathrm{Kv}}} = 0.0203 + 0.138 \cdot e^{(-(V_{\mathrm{Mfb}}+20.0)/25.9)^2}$$

$$\frac{ds_{\mathrm{Kv}}}{dt} = \frac{\bar{s}_{\mathrm{Kv}} - s_{\mathrm{Kv}}}{\tau_{s_{\mathrm{Kv}}}}$$

$$\tau_{s_{\mathrm{Kv}}} = 1.574 + 5.268 \cdot e^{(-(V_{\mathrm{Mfb}}+23.0)/22.7)^2}$$

$$E_{\mathrm{K,Mfb}} = \frac{\mathrm{RT}}{\mathrm{F}} \log \frac{[\mathrm{K}^+]_{\mathrm{c,Mfb}}}{[\mathrm{K}^+]_{\mathrm{i,Mfb}}}$$

Table 14. Time-independent inward-rectifying K+ current: $I_{\mathrm{K1\_Mfb}}$

$$I_{\mathrm{K1\_Mfb}} = \bar{g}_{\mathrm{K1,Mfb}} \left(\alpha_{\mathrm{K1}} / (\alpha_{\mathrm{K1}} + \beta_{\mathrm{K1}})\right) \left(V_{\mathrm{Mfb}} - E_{\mathrm{K,Mfb}}\right)$$

$$\alpha_{\mathrm{K1}} = 0.1\left(1.0 + e^{0.06(V_{\mathrm{Mfb}} - E_{\mathrm{K}} - 200.0)}\right)^{-1}$$

$$\beta_{\mathrm{K1}} = \frac{3.0 \cdot e^{0.0002(V_{\mathrm{Mfb}} - E_{\mathrm{K}} + 100.0)} + e^{0.1(V_{\mathrm{Mfb}} - E_{\mathrm{K}} + 10.0)}}{1.0 + e^{-0.5(V_{\mathrm{Mfb}} - E_{\mathrm{K}})}}$$

$$E_{\mathrm{K,Mfb}} = \frac{\mathrm{RT}}{\mathrm{F}} \log \frac{[\mathrm{K}^+]_{\mathrm{c,Mfb}}}{[\mathrm{K}^+]_{\mathrm{i,Mfb}}}$$

Table 15. Na$^+$-K$^+$ pump current: $I_{\mathrm{NaK\_Mfb}}$

$$I_{\text{NaK\_Mfb}} = \bar{I}_{\text{NaK,Mfb}} \cdot \frac{[\text{K}^+]_{\text{c,Mfb}}}{[\text{K}^+]_{\text{c,Mfb}} + k_{\text{mK}}} \cdot \frac{{[\text{Na}^+]_{\text{i,Mfb}}}^{1.5}}{{[\text{Na}^+]_{\text{i,Mfb}}}^{1.5} + {k_{\text{mNa}}}^{1.5}} \cdot \frac{V_{\text{Mfb}} + 150.0}{V_{\text{Mfb}} + 200.0}$$

Table 16. Background inward current: $I_{\text{B,Na\_Mfb}}$

$$I_{\text{B,Na\_Mfb}} = \bar{g}_{\text{B,Na,Mfb}}\left(V_{\text{Mfb}} - E_{\text{Na,Mfb}}\right)$$

$$E_{\text{Na,Mfb}} = \frac{\text{RT}}{\text{F}}\log\frac{[\text{Na}^+]_{\text{c,Mfb}}}{[\text{Na}^+]_{\text{i,Mfb}}}$$

Table 17. Na^+ current: $I_{\text{Na\_Mfb}}$

$$I_{\text{Na\_Mfb}} = \bar{g}_{\text{Na,Mfb}} m_{\text{Mfb}} j_{\text{Mfb}}^{0.12}\left(V_{\text{Mfb}} - E_{\text{Na,Mfb}}\right)$$

$$\bar{m}_{\text{Mfb}} = \frac{-0.0102 - 1.0063}{1.0 + e^{(V_{\text{Mfb}}+42.1)/10.53}} + 1.0063 \qquad \bar{j}_{\text{Mfb}} = \frac{1.04 - 0.004}{1.0 + e^{(V_{\text{Mfb}}+84.82)/9.4}} + 0.004$$

$$\frac{dm_{\text{Mfb}}}{dt} = \frac{\bar{m}_{\text{Mfb}} - m_{\text{Mfb}}}{\tau_{m_{\text{Mfb}}}} \qquad \frac{dj_{\text{Mfb}}}{dt} = \frac{\bar{j}_{\text{Mfb}} - j_{\text{Mfb}}}{\tau_{j_{\text{Mfb}}}}$$

$$\tau_{m_{\text{Mfb}}} = \frac{1}{\alpha_{m_{\text{Mfb}}} + \beta_{m_{\text{Mfb}}}} \qquad \tau_{j_{\text{Mfb}}} = \frac{1}{\alpha_{j_{\text{Mfb}}} + \beta_{j_{\text{Mfb}}}}$$

$$\alpha_{m_{\text{Mfb}}} = \begin{cases} 0.0077\dfrac{V_{\text{Mfb}} + 68.19}{1.0 - e^{-0.18(V_{\text{Mfb}}+68.19)}} \\ 0.0433, \quad \text{if } V_{\text{Mfb}} = -68.19 \end{cases}$$

$$\beta_{m_{\text{Mfb}}} = 0.004e^{-V_{\text{Mfb}}/11.98}$$

$$\alpha_{j_{\text{Mfb}}} = \begin{cases} \left(-14.5e^{0.17V_{\text{Mfb}}} + 1.8e^{1.56V_{\text{Mfb}}}\right) \cdot \dfrac{V_{\text{Mfb}} + 35.73}{1.0 + e^{3.31(V_{\text{Mfb}}+3.86)}} \\ 0, \quad \text{if } V_{\text{Mfb}} \geq -40.0 \end{cases}$$

$$\beta_{j_{\text{Mfb}}} = \begin{cases} 5.05 \times 10^{-5}\dfrac{e^{-0.0995V_{\text{Mfb}}}}{1.0 + e^{-0.01(V_{\text{Mfb}}-84.02)}} \\ 0.11\dfrac{e^{-4.13\times10^{-4}V_{\text{Mfb}}}}{1.0 + e^{-0.09(V_{\text{Mfb}}+42.44)}}, \text{ if } V_{\text{Mfb}} \geq -40.0 \end{cases}$$

$$E_{\text{Na,Mfb}} = \frac{\text{RT}}{\text{F}}\log\frac{[\text{Na}^+]_{\text{c,Mfb}}}{[\text{Na}^+]_{\text{i,Mfb}}}$$

Table 18. Mechano-gated channel mediated current: $I_{\text{MGC\_Mfb}}$

$$I_{\text{MGC\_Mfb}} = \bar{g}_{\text{MGC,Mfb}} \cdot \left(V_{\text{Mfb}} - E_{\text{MGC,Mfb}}\right)$$

Table 19. Intracellular ion concentrations: $[Na^+]_{i,Mfb}$, $[K^+]_{i,Mfb}$, and $[Ca^{2+}]_{i,Mfb}$

$$\text{Without } I_{\text{Na\_Mfb}}: \frac{d[\text{Na}^+]_{\text{i,Mfb}}}{dt} = -\frac{I_{\text{B,Na\_Mfb}} + 3I_{\text{NaK\_Mfb}}}{\text{Vol}_{\text{i,Mfb}}\text{F}}$$

$$\text{With } I_{\text{Na\_Mfb}}: \frac{d[\text{Na}^+]_{\text{i,Mfb}}}{dt} = -\frac{I_{\text{Na\_Mfb}} + I_{\text{B,Na\_Mfb}} + 3I_{\text{NaK\_Mfb}}}{\text{Vol}_{\text{i,Mfb}}\text{F}}$$

$$\frac{d[\mathrm{K}^+]_{\mathrm{i,Mfb}}}{dt} = -\frac{I_{\mathrm{K1\_Mfb}} + I_{\mathrm{Kv\_Mfb}}}{\mathrm{Vol}_{\mathrm{i,Mfb}}\mathrm{F}}$$

Mfb-M electrical coupling

Table 20. Transmembrane potential of myocyte and Mfb

$$\frac{dV_{\mathrm{M}}}{dt} = -\frac{1}{\mathrm{C}_{\mathrm{m,M}}}\left(I_{\mathrm{M}}(V_{\mathrm{M}},t) + \sum_{\mathrm{i=1}}^{\mathrm{n}} G_{\mathrm{gap}}\left(V_{\mathrm{M}} - V_{\mathrm{Mfb,i}}\right)\right)$$

$$\frac{dV_{\mathrm{Mfb,i}}}{dt} = -\frac{1}{\mathrm{C}_{\mathrm{m,Mfb}}}\left(I_{\mathrm{Mfb,i}}\left(V_{\mathrm{Mfb,i}},t\right) + G_{\mathrm{gap}}\left(V_{\mathrm{Mfb,i}} - V_{\mathrm{M}}\right)\right)$$

Without I_{SAC}: $I_{\mathrm{M}}(V_{\mathrm{M}},t) =$

$$I_{\mathrm{Na}} + I_{\mathrm{CaL}} + I_{\mathrm{t}} + I_{\mathrm{Kur}} + I_{\mathrm{K1}} + I_{\mathrm{K,r}} + I_{\mathrm{K,s}} + I_{\mathrm{B,Na}} + I_{\mathrm{B,Ca}} + I_{\mathrm{NaK}} + I_{\mathrm{CaP}} + I_{\mathrm{NaCa}} - I_{\mathrm{Stim}}$$

With I_{SAC}: $I_{\mathrm{M}}(V_{\mathrm{M}},t) =$

$$I_{\mathrm{Na}} + I_{\mathrm{CaL}} + I_{\mathrm{t}} + I_{\mathrm{Kur}} + I_{\mathrm{K1}} + I_{\mathrm{K,r}} + I_{\mathrm{K,s}} + I_{\mathrm{B,Na}} + I_{\mathrm{B,Ca}} + I_{\mathrm{NaK}} + I_{\mathrm{CaP}} + I_{\mathrm{NaCa}} + I_{\mathrm{SAC}} - I_{\mathrm{Stim}}$$

Without $I_{\mathrm{Na\_Mfb}}$ and $I_{\mathrm{MGC\_Mfb}}$: $I_{\mathrm{Mfb,i}}\left(V_{\mathrm{Mfb,i}},t\right) = I_{\mathrm{Kv\_Mfb}} + I_{\mathrm{K1\_Mfb}} + I_{\mathrm{NaK\_Mfb}} + I_{\mathrm{B,Na\_Mfb}}$

With $I_{\mathrm{Na\_Mfb}}$ and $I_{\mathrm{MGC\_Mfb}}$: $I_{\mathrm{Mfb,i}}\left(V_{\mathrm{Mfb,I}},t\right) =$

$$I_{\mathrm{Kv\_Mfb}} + I_{\mathrm{K1\_Mfb}} + I_{\mathrm{NaK\_Mfb}} + I_{\mathrm{B,Na\_Mfb}} + I_{\mathrm{Na\_Mfb}} + I_{\mathrm{MGC\_Mfb}}$$

Table 21. Parameter values

$[\mathrm{Na}^+]_{\mathrm{b}}$ = 130.0 mmol/L	k_{CaP} = 0.0002 mmol/L
$[\mathrm{K}^+]_{\mathrm{b}}$ = 5.4 mmol/L	k_{NaCa} = 0.0374842 pA/(mmol/L)^4
$[\mathrm{Ca}^{2+}]_{\mathrm{b}}$ = 1.8 mmol/L	γ = 0.45
$[\mathrm{Mg}^{2+}]_{\mathrm{i}}$ = 2.5 mmol/L	d_{NaCa} = 0.0003 (mmol/L)$^{-4}$
$E_{\mathrm{Ca,app}}$ = 60.0 mV	$\bar{I}_{\mathrm{up}}$ = 2800.0 pA
k_{Ca} = 0.025 mmol/L	k_{cyca} = 0.0003 mmol/L
R = 8314.0 mJ/molK	K_{srca} = 0.5 mmol/L
T = 306.15 K	K_{xcs} = 0.4
F = 96487.0 C/mol	τ_{tr} = 0.01 s
$\mathrm{C}_{\mathrm{m,M}}$ = 0.05 nF	α_{rel} = 200000.0 pA L/mmol
$\mathrm{Vol}_{\mathrm{i}}$ = 0.005884 nL	$k_{\mathrm{rel,i}}$ = 0.0003 mmol/L
$\mathrm{Vol}_{\mathrm{c}}$ = 0.136$\mathrm{Vol}_{\mathrm{i}}$	$k_{\mathrm{rel,d}}$ = 0.003 mmol/L
$\mathrm{Vol}_{\mathrm{d}}$ = 0.02$\mathrm{Vol}_{\mathrm{i}}$	r_{recov} = 0.815 s^{-1}
$\mathrm{Vol}_{\mathrm{rel}}$ = 0.0000441 nL	K_{SAC} = 100
$\mathrm{Vol}_{\mathrm{up}}$ = 0.0003969 nL	α_{SAC} = 3
τ_{Na} = 14.3 s	λ = 1.2
τ_{K} = 10.0 s	$\mathrm{C}_{\mathrm{m,Mfb}}$ = 6.3 pF
τ_{Ca} = 24.7 s	$\mathrm{Vol}_{\mathrm{i,Mfb}}$ = 0.00137 nL

τ_{dt} = 0.01 s	$[Na^+]_{c,Mfb}$ = 130.011 mmol/L
$\bar{I}_{NaK}$ = 68.55 pA	$[K^+]_{c,Mfb}$ = 5.3581 mmol/L
$k_{NaK,K}$ = 1.0 mmol/L	k_{mK} = 1.0 mmol
$k_{NaK,Na}$ = 11.0 mmol/L	k_{mNa} = 11.0 mmol
$\bar{I}_{CaP}$ = 4.0 pA	$\bar{I}_{NaK,Mfb}$ = 10.36 pA

Table 22. Maximum conductance values

P_{Na} = 0.0018 nL/s	$\bar{g}_{B,Ca}$ = 0.078681 nS
$\bar{g}_{CaL}$ = 6.75 nS	G_{SAC} = 0.015 μm/s
$\bar{g}_{t}$ = 8.25 nS	$\bar{g}_{Kv,Mfb}$ = 1.575 nS
$\bar{g}_{Kur}$ = 2.25 nS	$\bar{g}_{K1,Mfb}$ = 3.038 nS
$\bar{g}_{K,s}$ = 1.0 nS	$\bar{g}_{B,Na,Mfb}$ = 0.05985 nS
$\bar{g}_{K,r}$ = 0.5 nS	$\bar{g}_{Na,Mfb}$ = 0.756 nS
$\bar{g}_{K1}$ = 3.1 nS	$\bar{g}_{MGC,Mfb}$ = 0.043 nS
$\bar{g}_{B,Na}$ = 0.060599 nS	

Table 23. Initial conditions

V_M = -74.2525 mV	s_{Kur} = 0.9673
$[Na^+]_c$ = 130.0221 mmol/L	$n = 4.374\times10^{-3}$
$[K^+]_c$ = 5.5602 mmol/L	$p_a = 5.3\times10^{-5}$
$[Ca^{2+}]_c$ = 1.8158 mmol/L	F_1 = 0.4701
$[Na^+]_i$ = 8.5168 mmol/L	F_2 = 0.0028
$[K^+]_i$ = 129.486 mmol/L	O = 1.382
$[Ca^{2+}]_i = 6.5\times10^{-5}$ mmol/L	O_C = 0.0268
$[Ca^{2+}]_d = 7.1\times10^{-5}$ mmol/L	O_{Calse} = 0.4315
$[Ca^{2+}]_{up}$ = 0.6492 mmol/L	O_{TC} = 0.0129
$[Ca^{2+}]_{rel}$ = 0.6326 mmol/L	O_{TMgC} = 0.1904
$m = 3.289\times10^{-3}$	O_{TMgMg} = 0.7145
h_1 = 0.8772	$[K^+]_{i,Mfb}$ = 129.4349 mmol/L
h_2 = 0.8739	$[Na^+]_{i,Mfb}$ = 8.5547 mmol/L
$d_L = 1.4\times10^{-5}$	V_{Mfb} = -47.75 mV
f_{L1} = 0.9986	r_{Kv} = 0.0743
f_{L2} = 0.9986	s_{Kv} = 0.9717
$r = 1.089\times10^{-3}$	$m_{Mfb} = 3.0\times10^{-3}$
s = 0.9486	j_{Mfb} = 0.9989
$r_{Kur} = 3.67\times10^{-4}$	

Glossary

	**Myocyte**	Vol_i	Total cytosolic volume
I_{Na}	Na^+ current	Vol_d	Volume of the diffusion-restricted subsarcolemmal space
I_{CaL}	L-type Ca^{2+} current	Vol_{up}	Volume of the sarcoplasmic reticulum uptake compartment

I_t	Transient outward K^+ current
I_{Kur}	Sustained outward K^+ current
$I_{K,s}$	Slow delayed rectifier K^+ current
$I_{K,r}$	Rapid delayed rectifier K^+ current
I_{K1}	Inwardly rectifying K^+ current
$I_{B,Na}$	Background Na^+ current
$I_{B,Ca}$	Background Ca^{2+} current
I_{NaK}	Na^+-K^+ pump current
I_{CaP}	Sarcolemmal Ca^{2+} pump current
I_{NaCa}	Na^+-Ca^{2+} exchange current
I_{di}	Ca^{2+} diffusion current from the diffusion-restricted subsarcolemmal space to the cytosol
I_{up}	Sarcoplasmic reticulum Ca^{2+} uptake current
I_{tr}	Sarcoplasmic reticulum Ca^{2+} translocation current (from uptake to release compartment)
I_{rel}	Sarcoplasmic reticulum Ca^{2+} release current
$[Na^+]_b$	Na^+ concentration in bulk (bathing) medium
$[K^+]_b$	K^+ concentration in bulk (bathing) medium
$[Ca^{2+}]_b$	Ca^{2+} concentration in bulk (bathing) medium
$[Na^+]_c$	Na^+ concentration in the extracellular cleft space
$[K^+]_c$	K^+ concentration in the extracellular cleft space
$[Ca^{2+}]_c$	Ca^{2+} concentration in the extracellular cleft space
$[Na^+]_i$	Na^+ concentration in the intracellular medium
$[K^+]_i$	K^+ concentration in the intracellular medium
$[Ca^{2+}]_i$	Ca^{2+} concentration in the intracellular medium
$[Mg^{2+}]_i$	Mg^{2+} concentration in the intracellular medium
$[Ca^{2+}]_d$	Ca^{2+} concentration in the restricted subsarcolemmal space
$[Ca^{2+}]_{up}$	Ca^{2+} concentration in the sarcoplasmic reticulum uptake compartment
$[Ca^{2+}]_{rel}$	Ca^{2+} concentration in the sarcoplasmic reticulum release compartment
E_{Na}	Equilibrium (Nernst) potential for Na^+
E_K	Equilibrium (Nernst) potential for K^+
E_{Ca}	Equilibrium (Nernst) potential for Ca^{2+}
$E_{Ca,app}$	Apparent reversal potential for I_{CaL}
P_{Na}	Permeability for I_{Na}
$\bar{g}_{CaL}$	Maximum conductance for I_{CaL}
$\bar{g}_t$	Maximum conductance for I_t
$\bar{g}_{Kur}$	Maximum conductance for I_{Kur}
$\bar{g}_{K,s}$	Maximum conductance for $I_{K,s}$
$\bar{g}_{K,r}$	Maximum conductance for $I_{K,r}$

Vol_{rel}	Volume of the sarcoplasmic reticulum release compartment
τ_{Na}, τ_K, τ_{Ca},	Time constant of diffusion of Na^+, K^+, and Ca^{2+} from the bulk medium to the extracellular cleft space
τ_{di}	Time constant of diffusion from the restricted subsarcolemmal space to the cytosol
$\bar{I}_{NaK}$	Maximum Na^+-K^+ pump current
$k_{NaK,K}$	Half-maximum K^+ binding concentration for I_{NaK}
$k_{NaK,Na}$	Half-maximum Na^+ binding concentration for I_{NaK}
$\bar{I}_{CaP}$	Half-maximum Ca2+ binding concentration for I_{CaP}
k_{NaCa}	Scaling factor for I_{NaCa}
γ	Position of energy barrier controlling voltage dependence of I_{NaCa}
d_{NaCa}	Denominator constant for I_{NaCa}
$\bar{I}_{up}$	Maximum sarcoplasmic reticulum uptake current
k_{cyca}	Half-maximum binding concentration for $[Ca^{2+}]_i$ to I_{up}
k_{srca}	Half-maximum binding concentration for $[Ca^{2+}]_{up}$ to I_{up}
K_{xcs}	Ratio of forward to back reactions for I_{up}
τ_{tr}	Time constant of diffusion of Ca^{2+} from sarcoplasmic reticulum uptake to release compartment
α_{rel}	Scaling factor for I_{rel}
$k_{rel,i}$	Half-activation $[Ca^{2+}]_i$ for I_{rel}
$k_{rel,d}$	Half-activation $[Ca^{2+}]_d$ for I_{rel}
k_{recov}	Recovery rate constant for the sarcoplasmic reticulum release channel
I_{SAC}	Stretch-activated current
$I_{SAC,Na}$	Na^+ contributes to I_{sac}
$I_{SAC,K}$	K^+ contributes to I_{sac}
$I_{SAC,Ca}$	Ca^{2+} contributes to I_{sac}
$\bar{P}_{Na}$	Relative permeability to Na^+
$\bar{P}_K$	Relative permeability to K^+
$\bar{P}_{Ca}$	Relative permeability to Ca^{2+}
z_{Na}	Na^+ valence
z_K	K^+ valence
z_{Ca}	Ca^{2+} valence
g_{SAC}	Conductance for I_{sac}
G_{SAC}	Maximum conductance for I_{sac}
K_{SAC}	Parameter to define the amount of current when the cell is not stretched
α_{SAC}	Parameter to describe the sensitivity to stretch
λ	Stretch ratio
	**Myofibroblast**
I_{Kv_Mfb}	Time- and voltage-dependent K^+ current
I_{K1_Mfb}	Inward-rectifying K^+ current

$\bar{g}_{K1}$	Maximum conductance for I_{K1}
$\bar{g}_{B,Na}$	Maximum conductance for $I_{B,Na}$
$\bar{g}_{B,Ca}$	Maximum conductance for $I_{B,Ca}$
m	Activation gating variable for I_{Na}
h_1, h_2	Fast and slow inactivation gating variables for I_{Na}
d_L	Activation gating variable for I_{CaL}
f_{L1}, f_{L2}	Fast and slow inactivation gating variables for I_{CaL}
f_{Ca}	$[Ca^{2+}]_d$-dependent ratio of fast (f_{L1}) to slow (f_{L2}) inactivation of I_{CaL}
k_{Ca}	Half-maximum Ca^{2+} binding concentration for f_{Ca}
r	Activation gating variable for I_t
s	Inactivation gating variable for I_t
s_1, s_2	Rapidly and slowly recovering inactivation gating variables for I_t
r_{Kur}	Activation gating variable for I_{Kur}
s_{Kur}	Inactivation gating variable for I_{Kur}
n	Activation gating variable for $I_{K,s}$
p_a	Activation gating variable for $I_{K,r}$
p_i	Inactivation gating variable (instantaneous) for $I_{K,r}$
$\bar{m}, \bar{h}_1, \cdots$	Steady-state value of m, h_1, etc
F_1	Relative amount of "inactive precursor" in the I_{rel} formulation
F_2	Relative amount of "activator" in the I_{rel} formulation
τ_m	Activation time constant for I_{Na}
τ_{h_1}, τ_{h_2}	Fast and slow inactivation time constants for I_{Na}
τ_{d_L}	Activation time constant for I_{CaL}
$\tau_{f_{L1}}, \tau_{f_{L2}}$	Fast and slow inactivation time constants for I_{CaL}
τ_r	Activation time constant for I_t
τ_s	Inactivation time constant for I_t
$\tau_{r_{Kur}}$	Activation time constant for I_{Kur}
$\tau_{s_{Kur}}$	Inactivation time constant for I_{Kur}
τ_n	Activation time constant for $I_{K,s}$
τ_{p_a}	Activation time constant for $I_{K,r}$
O	Buffer occupancy
O_C	Fractional occupancy of the calmodulin buffer by Ca^{2+}
O_{TC}	Fractional occupancy of the troponin-Ca^{2+} buffer by Ca^{2+}
O_{TMgC}	Fractional occupancy of the troponin-Mg^{2+} buffer by Ca^{2+}
O_{TMgMg}	Fractional occupancy of the troponin-Mg^{2+} buffer by Mg^{2+}
O_{Calse}	Fractional occupancy of the calsequestrin buffer (in the sarcoplasmic reticulum release compartment) by Ca^{2+}
R	Universal gas constant
T	Absolute temperature
F	Faraday's constant
$C_{m,M}$	Membrane capacitance

I_{NaK_Mfb}	Na^+-K^+ pump current
I_{B,Na_Mfb}	Background Na^+ current
I_{Na_Mfb}	Na^+ current
I_{MGC_Mfb}	Mechano-gated current
$[Na^+]_{c,Mfb}$	Na^+ concentration in the extracellular cleft space
$[K^+]_{c,Mfb}$	K^+ concentration in the extracellular cleft space
$[Na^+]_{i,Mfb}$	Na^+ concentration in the intracellular medium
$[K^+]_{i,Mfb}$	K^+ concentration in the intracellular medium
$E_{Na,Mfb}$	Equilibrium (Nernst) potential for Na^+
$E_{K,Mfb}$	Equilibrium (Nernst) potential for K^+
$E_{MGC,Mfb}$	Equilibrium (Nernst) potential for ion through mechano-gate channels
$\bar{g}_{Kv,Mfb}$	Maximum conductance for I_{Kv_Mfb}
$\bar{g}_{K1,Mfb}$	Maximum conductance for I_{K1_Mfb}
$\bar{g}_{B,Na,Mfb}$	Maximum conductance for I_{b,Na_Mfb}
$\bar{g}_{Na,Mfb}$	Maximum conductance for I_{Na_Mfb}
$\bar{g}_{MGC,Mfb}$	Maximum conductance for I_{MGC_Mfb}
r_{Kv}	Activation gating variable for I_{Kv_Mfb}
s_{Kv}	Inactivation gating variable for $I_{Kv\ Mfb}$
$\tau_{r_{Kv}}$	Activation time constant for I_{Kv_Mfb}
$\tau_{s_{Kv}}$	Inactivation time constant for I_{Kv_Mfb}
α_{K1}, β_{K1}	Fractional open probability of the I_{K1_Mfb} channel
$\bar{I}_{NaK,max}$	Maximum Na^+-K^+ pump current
k_{mK}	Half-maximum K^+ binding concentration for I_{NaK}
k_{mNa}	Half-maximum Na^+ binding concentration for I_{NaK}
m_{Mfb}	Activation gating variable for I_{Na_Mfb}
j_{Mfb}	Inactivation gating variable for $I_{Na\ Mfb}$
$\tau_{m_{Mfb}}$	Activation time constant for I_{Na_Mfb}
$\tau_{j_{Mfb}}$	Inactivation time constant for I_{Na_Mfb}
$\alpha_{m_{Mfb}}, \alpha_{j_{Mfb}}, \beta_{m_{Mfb}}, \beta_{j_{Mfb}}$	Extrapolated rate coefficients
$E_{Na,Mfb}$	Equilibrium (Nernst) potential for Na^+
$E_{K,Mfb}$	Equilibrium (Nernst) potential for K^+
$E_{MGC,Mfb}$	Equilibrium (Nernst) potential for ion through mechano-gate channels
$\bar{r}_{Kv}, \bar{s}_{Kv}, \cdots$	Steady-state value of r_{Kv}, s_{Kv}, etc
$C_{m,Mfb}$	Membrane capacitance
V_{Mfb}	Membrane voltage

Mfb-M coupling

G_{gap}	Gap junctional conductance between Mfb and myocyte
n	Number of Mfbs per myocyte
I_M	Net membrane current of the myocyte
I_{Mfb}	Net membrane current of the Mfb

V_M	Membrane voltage
Vol_c	Volume of the extracellular cleft space

References

[1] M. M. Maleckar, J. L. Greenstein, W. R. Giles, and N. A. Trayanova, "K+ current changes account for the rate dependence of the action potential in the human atrial myocyte," *Am J Physiol Heart Circ Physiol*, vol. 297, pp. H1398-410, 2009.

[2] A. Nygren, C. Fiset, L. Firek, J. W. Clark, D. S. Lindblad, R. B. Clark, and W. R. Giles, "Mathematical model of an adult human atrial cell: the role of K+ currents in repolarization," *Circ Res*, vol. 82, pp. 63-81, 1998.

[3] N. H. Kuijpers, H. M. ten Eikelder, P. H. Bovendeerd, S. Verheule, T. Arts, and P. A. Hilbers, "Mechanoelectric feedback leads to conduction slowing and block in acutely dilated atria: a modeling study of cardiac electromechanics," *Am J Physiol Heart Circ Physiol*, vol. 292, pp. H2832-53, 2007.

[4] D. Kim, "Novel cation-selective mechanosensitive ion channel in the atrial cell membrane," *Circ Res*, vol. 72, pp. 225-31, 1993.

[5] K. A. MacCannell, H. Bazzazi, L. Chilton, Y. Shibukawa, R. B. Clark, and W. R. Giles, "A mathematical model of electrotonic interactions between ventricular myocytes and fibroblasts," *Biophysical Journal*, vol. 92, pp. 4121-4132, 2007.

[6] A. Chatelier, A. Mercier, B. Tremblier, O. Theriault, M. Moubarak, N. Benamer, P. Corbi, P. Bois, M. Chahine, and J. F. Faivre, "A distinct de novo expression of Nav1.5 sodium channels in human atrial fibroblasts differentiated into myofibroblasts," *J Physiol*, vol. 590, pp. 4307-19, 2012.

[7] A. Kamkin, S. Kirischuk, and I. Kiseleva, "Single mechano-gated channels activated by mechanical deformation of acutely isolated cardiac fibroblasts from rats," *Acta Physiol (Oxf)*, vol. 199, pp. 277-92, 2010.
